# Hyperkalemia and Electrocardiogram Manifestations in End-Stage Renal Disease

**DOI:** 10.3390/ijerph192316140

**Published:** 2022-12-02

**Authors:** Zubaid Rafique, Bryan Hoang, Heba Mesbah, Ryan Pappal, Frank W. Peacock, Raul Juarez-Vela, Lukasz Szarpak, Dick C. Kuo

**Affiliations:** 1 Henry JN Taub Department of Emergency Medicine, Baylor College of Medicine, Houston, TX 77030, USA; 2 McGovern Medical School, University of Texas Health Science Center at Houston, Houston, TX 77004, USA; 3 Washington University School of Medicine in St. Louis, St. Louis, MO 63110, USA; 4 Group in Research in Care (GRUPAC), Department of Nursing, University of La Rioja, 93-103 Logrono, Spain

**Keywords:** hyperkalemia, potassium, electrocardiogram, dysrhythmia

## Abstract

Hyperkalemia is one of the more common acute life-threatening metabolic emergencies. The aim of our study is to determine the correlation and accuracy of abnormal ECG parameters as a function of serum potassium concentration in the end-stage renal disease (ESRD) population. We performed a retrospective chart review of emergency department patients presenting with ESRD and receiving emergent hemodialysis treatment. A total of 96 patients, each with five independent ED visits, provided 480 sets of ECGs and electrolytes. Of these, four ECGs were excluded for inability to interpret, leaving a total of 476 patient encounters that met all inclusion criteria. Linear regression analysis on the limited data set for serum potassium versus T/R in V2, V3, and V4, PR, and QRS found weak correlations (r^2^ = 0.02 to 0.12) with statistical significance <0.05 level for T/R in V2, V3, and V4. In summary, we found that a QRS duration of 120 ms or greater is most predictive of hyperkalemia in the ESRD population. On the other hand, T/R ratio, PR interval and QRS duration have poor correlations with serum potassium and are not predictive of hyperkalemia in patients with ESRD.

## 1. Introduction

Hyperkalemia is a common electrolyte disturbance in patients with chronic kidney disease (CKD) and end-stage renal disease (ESRD) [1,2,3,4,5,6,7,8]. With the aging population in the U.S. and the growing number of patients with DM, CHF and CKD, presentations for hyperkalemia are increasing [2,9,10,11,12,13,14]. Although hyperkalemia may cause life threatening arrhythmia, there is a lack of consensus on what constitutes a severe versus a mild potassium elevation and the role of the ECG in its diagnosis and treatment [3,15,16,17,18]. Recent observational studies underscore the clinical significance of hyperkalemia by associating it with considerable in- and out-of-hospital mortality [13,19,20] and highlight the importance of its recognition and urgent correction. 

Clinical manifestations of hyperkalemia usually involve nonspecific symptoms such as generalized lethargy, nausea, abdominal pain, and diarrhea [21,22]. In severe hyperkalemia, defined as ≥6.50 mEq/L, muscle paralysis and cardiac arrhythmias may develop [22,23]. In a normal cardiac rhythm, myocardial depolarization is dependent upon a concentration gradient created by the sodium–potassium ATPase pump. Impairment of these electrophysiological functions by hyperkalemia can lead to abnormal rhythm. Elevated extracellular potassium diminishes its concentration gradient across the cellular membrane and decreases the overall resting membrane potential [24]. The decrease in resting membrane potential desensitizes sodium channels, which blunts the effect of sodium-dependent depolarization, and produces a prolonging effect in membrane depolarization [24]. In addition, the electrolyte imbalance across a membrane potential can manifest as ECG changes that include tenting of T-wave amplitudes, PR interval lengthening, disappearance of the P-wave, loss of R-waves, delayed conduction, widening of QRS complex, ST segment elevation, and a sine-like wave pattern [25,26,27,28,29,30,31]. Although these ECG changes are a characteristic of hyperkalemia, they do not always occur because of the simultaneous interaction between potassium and other electrolytes in the setting of complex metabolic derangements [26,30,31,32,33,34]. 

While hyperkalemia is commonly associated with ECG changes, the role of the ECG in the diagnosis and management of hyperkalemia in clinically stable patients has been questioned. Even though pathologic ECG changes may manifest in the setting of experimentally induced hyperkalemia, the reliability of this diagnosis is less clear and potentially inconsistent in the clinical environment [35,36]. 

It is, therefore, important to determine the reliability of abnormal ECG manifestations when used to detect cases of hyperkalemia. The aim of our study is to determine the correlation and accuracy of abnormal ECG parameters as a function of serum potassium concentration in the ESRD population.

## 2. Materials and Methods

We performed a retrospective chart review of emergency department patients presenting with ESRD and receiving emergent hemodialysis treatment at an academic inner city county hospital with an annual census of more than a hundred thousand patients. The study was approved by the institutional review board (approval no. IRB# H-34607) and patients were identified by electronic medical record query. The entry criteria included patients who received emergent hemodialysis (patients who did not have scheduled dialysis sessions and only received dialysis through emergency departments) between April 2012 and December 2014, and who had been on hemodialysis >3 months. Since patients made repeated visits and each visit had unique data, they were allowed to be enrolled up to five times. Patients were excluded if they had ECG of poor quality that could not be interpreted, or a baseline rhythm that precluded the accurate identification of QRS widening (e.g., right or left bundle branch block and paced rhythm) and left ventricular hypertrophy. Demographics, electrocardiograms, and concurrent electrolytes prior to receiving hemodialysis, were recorded. Electrolytes were measured using a point of care (POC) i-STAT system (Abbott iStat Inc., Princeton, NJ). Time dependent ECG parameters (e.g., PR and QRS length) were recorded from the ECG tracings (device generated intervals), while T-wave and R-wave amplitudes were manually measured by research coordinators. 

### Statistical Analysis

Demographics are presented using descriptive statistics. In order to assess the relationship between ECG parameter and serum potassium, a partial linear correlation was performed for evaluating T/R ratio in the precordial ECG leads V2, V3, and V4, while controlling for serum calcium and bicarbonate. 

In addition, ROC analyses were generated with calculated sensitivities, specificities, and positive and negative predictive values using cutoff thresholds of 0.75 for T-wave to R-wave ratio, 200 ms for PR interval, and 120 ms for QRS duration, where hyperkalemia was defined as [K+] ≥ 5.3 mEq/L. Optimal cutoff points were also calculated using the Youden Index. Outliers were removed based on Z-scores greater than 3 or less than −3. Due to the removal of outliers, certain analyses had different sample sizes. All analyses were performed using Microsoft Excel 2013 for Windows, version 15.0.5007.1000, MedCalc for Windows, version 18.0 (MedCalc Software, Ostend, Belgium), and SPSS (IBM Corp. Released 2013. IBM SPSS Statistics for Windows, Version 22.0. Armonk, NY, USA: IBM Corp). 

## 3. Results

A total of 96 patients, each with five independent ED visits, provided 480 sets of ECGs and electrolytes. Of these, four ECGs were excluded for inability to interpret, leaving a total of 476 patient encounters that met all inclusion and exclusion criteria. There were 57 male (59.4%) and 39 female (40.6%) patients with a mean (± SD) age of 46 (±15) years. The sample identified as 92.7% Hispanic, 3.1% Asian, and 4.2% African American. The prevalence of hyperkalemia was 89.6% on the first visit of each of 96 patients, and 89.5% of all 476 data points. The mean (± SD) serum potassium level was 6.2 ± 0.77 mEq/L, with a range of 3.4 to 8.4 mEq/L. Demographics, ECG characteristics, and mean (± SD) serum electrolytes values are presented in Table 1. 

Analyses were performed on two data sets: limited and complete. The limited data set comprised 96 ECG and electrolyte pairs from the initial visits of the 96 subjects enrolled in the study, while the complete set included all 476 visits.

**Figure 1:**
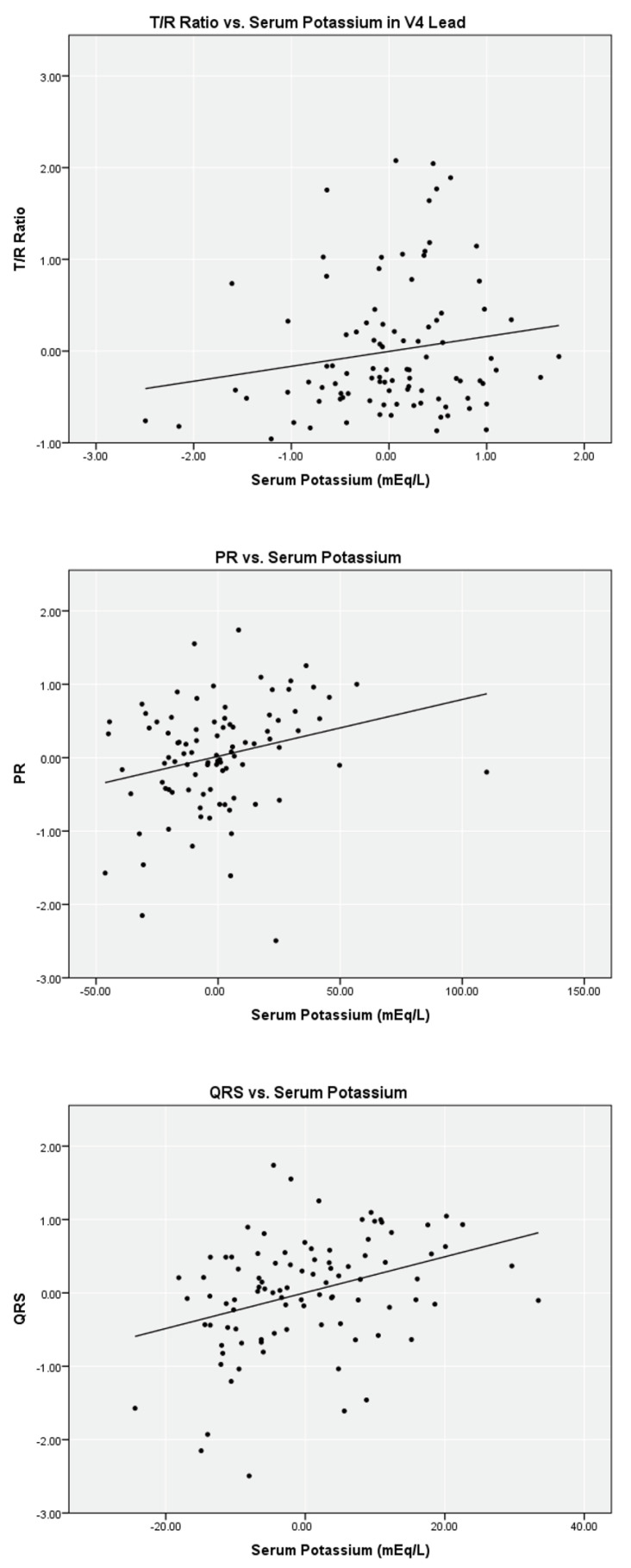
Partial linear correlation of serum potassium versus ECG parameters while controlling for ionized calcium and bicarbonate on limited data set (*n* = 96).

**Figure 2:**
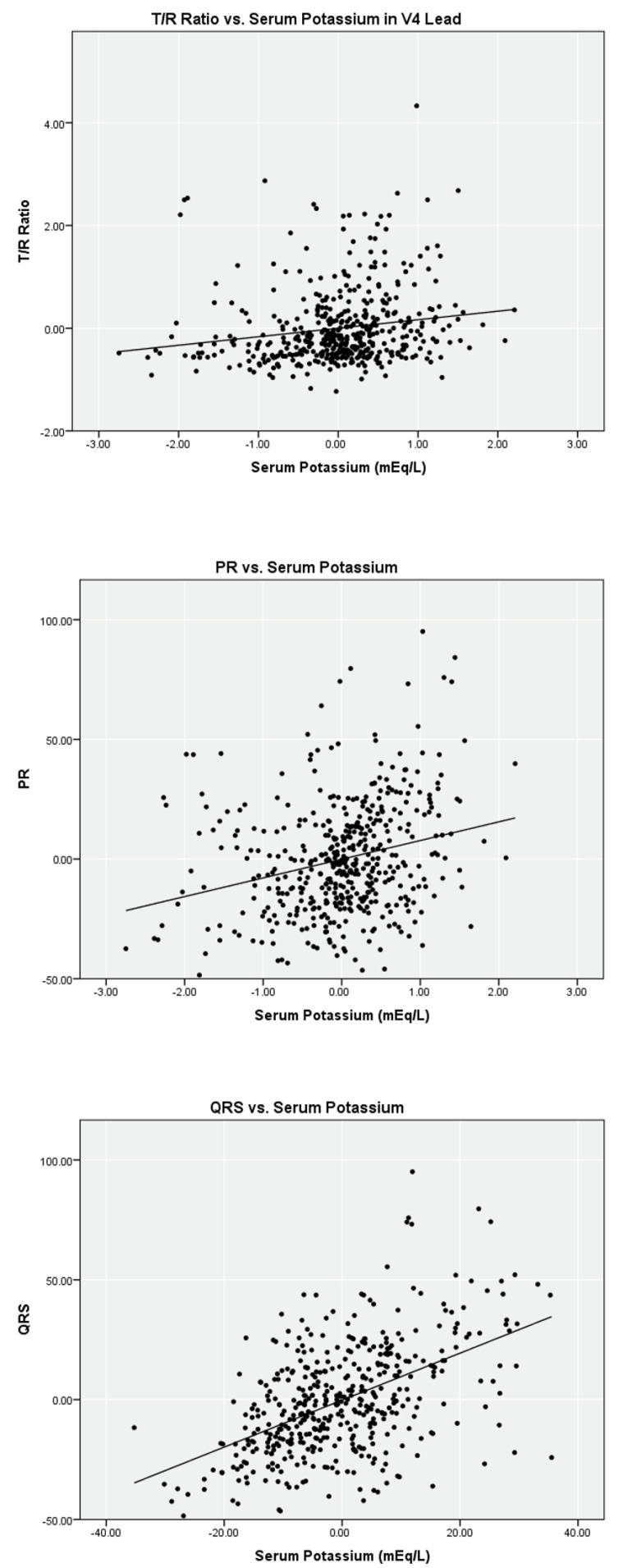
Partial linear correlation of serum potassium versus ECG parameters while controlling for ionized calcium and bicarbonate on complete data set (*n* = 476).

Linear regression analysis on the limited data set for serum potassium versus T/R in V2, V3, and V4, PR, and QRS found weak correlations (r2 = 0.02 to 0.12) with a statistical significance < 0.05 level for T/R in V2, V3, and V4 (Table 2). Scatter plots with fitted lines of T/R ratio in the V4 lead, PR, and QRS are shown in Figure 1. Similar analyses were performed with the complete data set. Regression analysis for T/R in V2, V3, and V4, PR, and QRS versus serum potassium also yielded weak correlation, and the scatter plots are shown in Figure 2. 

**Figure 3:**
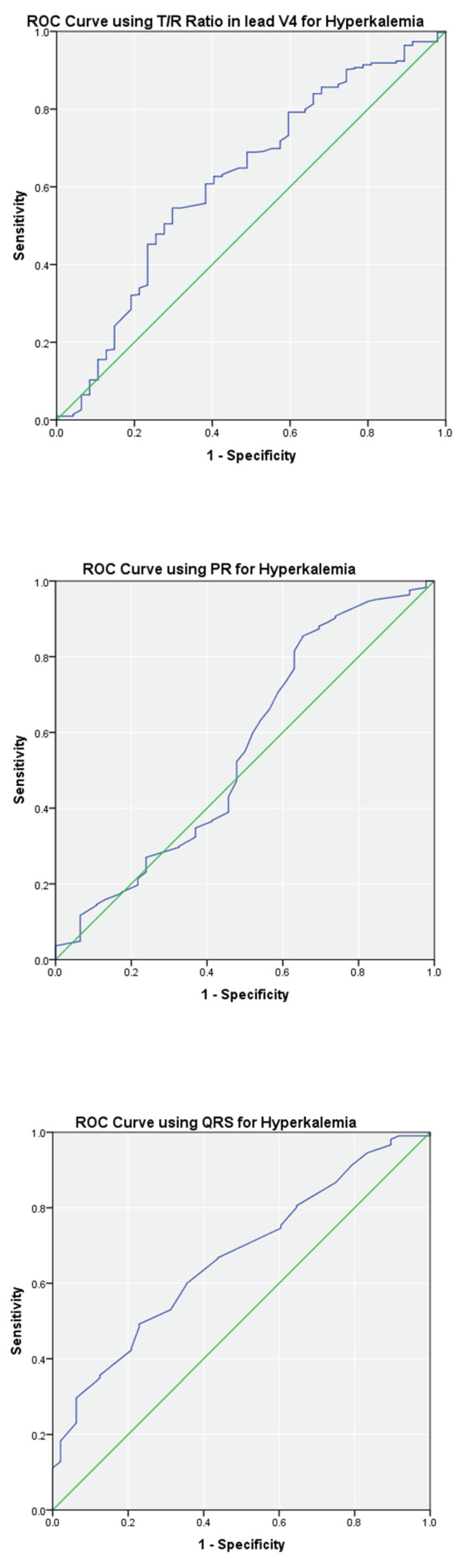
ROC curves of T/R ratio in V4, PR and QRS using the complete data set (*n* = 476). The blue line shows the performance of ECG parameter. The green line is for reference.

ROC analyses describing the ability of the T/R ratio in V2, V3, and V4, PR, and QRS to predict hyperkalemia are summarized in Table 3. The highest area under the curve (AUC) was noted for the T/R ratio of ≥0.75 in lead V2 (AUC = 0.80; *p* < 0.01) yielding a fair sensitivity of 0.68 and moderate specificity of 0.80. A specificity of 1.00 was noted for PR (≥200 ms) and QRS (≥120 ms), however the sensitivities were poor. Optimal cutoff thresholds for T/R in V2, T/R in V3, T/R in V4, PR, and QRS are shown in Table 3 with AUC ranging from 0.69 to 0.80 (*p* < 0.05). Similar analysis on the complete data set is summarized in Table 3. In general, the AUC decreased minimally without affecting the sensitivities and specificities. ROC curves for T/R in V4, PR, and QRS on the complete data set are shown in Figure 3. 

## 4. Discussion

Electrocardiograms are a cornerstone in the management of hyperkalemia. However, recent studies have questioned their predictive value and rendered their role unclear in hyperkalemia management [30,33,35,37,38,39]. Past studies that have investigated the relationship between ECG and hyperkalemia have been limited by the number of ECGs evaluated and parameters (i.e., peaked T-waves, QRS duration, etc.) analyzed. We performed the largest ECG analysis of patients presenting to the ED with hyperkalemia, to date. We found that the T/R ratio in V2, V3, and V4, PR interval, and QRS duration had poor correlations with serum potassium. QRS length had the best correlation with an adjusted R Square value of 0.09, signifying that only 9% of the change in QRS duration was explained by a change in potassium level. However, based on the ROC analysis, a QRS threshold ≥120 ms yielded a perfect specificity and a 100% positive predictive value for hyperkalemia, signifying that a QRS duration greater than 120 ms is a reliable ECG parameter to rule in hyperkalemia in the ESRD population. 

Furthermore, ROC analysis of the T/R ratio in V2, V3, and V4, PR interval, and QRS duration suggested a fair diagnostic performance in all the parameters but PR interval, with fair sensitivity and specificity. Using optimal cutoff thresholds based off the Youden Index for each of the ECG characteristics, we found a significant tradeoff between sensitivity and specificity. Although the diagnostic performances of the ECG parameters were statistically significant, due to the poor sensitivity and specificity, the clinical utility of these parameters was poor. However, it is plausible that a computer model utilizing multiple parameters simultaneously could predict hyperkalemia more reliably, as noted by Attia et al. [40].

Our results were similar to other studies that investigated the correlation between abnormal ECG manifestations and hyperkalemia and found that ECG parameters are unreliable in detecting hyperkalemia [30,33,35,37,38,39]. In particular, a study by Green and colleagues investigated the relationship between ECG and hyperkalemia in 145 patients with CKD, and determined that a T/R ratio of ≥0.75 yielded poor sensitivity but high specificity in identifying hyperkalemia. In another study by Aslam et al., ECG parameters from 74 ESRD patients were analyzed to conclude that hyperkalemia might not reliably cause ECG changes. On the contrary, our results were different from those published by Tarif et al. who found that the T/R ratio changes significantly between pre- and post-dialysis, implying that serum potassium is tightly correlated with ECG parameters. It is possible that potassium concentration dictates certain ECG parameters within the same patient, however, when evaluated as a group this difference might disappear as seen in multiple studies, including ours.

This study is more robust than previous studies because of several reasons. Firstly, the ECGs were collected simultaneously with electrolyte measurements, which assured tight data pairing. Secondly, ECGs with poor tracings, bundle branch blocks, left ventricular hypertrophy and paced rhythm were excluded to reduce confounders (e.g., interval prolongation due to structural abnormality versus elevated potassium). Thirdly, regression analysis accounted for calcium and bicarbonate, thus, reducing effects of other electrolytes on ECG parameters. Lastly, this is the largest study that evaluated multiple ECG parameters to investigate the relationship between hyperkalemia and ECGs.

### Limitations

There were several limitations to this study as well. Firstly, the study was specific to ESRD patients with chronic hyperkalemia and might not be applicable to patients with normal kidney function or those with acute hyperkalemia. Secondly, even though we controlled for serum calcium and bicarbonate levels in our regression model, it was possible that we missed other critical electrolytes, resulting in poor correlation. Thirdly, we analyzed multiple ECG parameters, but not every possible parameter (e.g., slope of T-wave or duration of T-wave). Lastly, our cohort comprised mostly of patients of Hispanic ethnicity, and, thus, generalizing the implication of our results to other ethnicities should be undertaken with caution. 

## 5. Conclusions

We analyzed 476 ECGs and found that a QRS duration of 120 ms or greater was most predictive of hyperkalemia in the ESRD population. On the other hand, T/R ratio, PR interval and QRS duration had poor correlations with serum potassium and, thus, lacked sufficient sensitivity to exclude the possibility of hyperkalemia in patients with ESRD.

## Figures and Tables

**Table 1:** Demographics, electrocardiogram (ECG) characteristics, and mean serum electrolytes.

Characteristic	Value (%)
Age, years	
Mean ± SD	46 ± 15
Gender	
Male	57
Female	39
Race (Ethnicity)	
Hispanic	89 (92.70)
Asian	3 (3.13)
Black	4 (4.17)
ECG Characteristics	
Normokalemia (K ≤ 5.3 mEq/L)	10 (10.40)
Hyperkalemia (K ≥ 5.3 mEq/L)	86 (89.60)
Patient Visits	476
Normokalemia (K ≤ 5.3 mEq/L)	50 (10.50)
Hyperkalemia (K ≥ 5.3 mEq/L)	426 (89.50)
Mean Serum Electrolytes ± SD	
Potassium	6.20 ± 0.77
Ionized Calcium	0.97 ± 0.15
Sodium	135.90 ± 7.56
Chloride	107 ± 5.28
Bicarbonate	20.30 ± 4.32
Glucose	110.30 ± 49.00
Blood Urea Nitrogen	104.80 ± 29.40
Creatinine	12.40 ± 7.69

Legend: K = serum potassium.

**Table 2:** Partial linear correlations of T/R ratio by lead, PR, and QRS vs. serum potassium.

Sample Size (N)	ECG Characteristic	Correlation Coefficient	Adjusted R Square	*p*-Value (2-Tailed)
	T/R Ratio			
	Lead			
n = 95	V2	0.23	0.04	0.04
n = 94	V3	0.17	0.02	0.02
n = 95	V4	0.17	0.02	0.02
n = 92	PR	0.26	0.06	0.06
n = 95	QRS	0.35	0.12	0.12
	T/R Ratio			
	Lead			
n = 466	V2	0.21	0.04	<0.01
n = 472	V3	0.17	0.03	<0.01
n = 465	V4	0.17	0.03	<0.01
n = 457	PR	0.29	0.06	<0.01
n = 469	QRS	0.31	0.09	<0.01

Legend: values of N are slightly different because outliers were excluded using Z-scores.

**Table 3:** Receiver operating characteristic (ROC) analysis for T/R ratio by lead, PR, and QRS.

Sample Size (N)	ECG Test Variable	Cutoff Threshold (Optimal)	Sensitivity (Optimal)	Specificity (Optimal)	Positive Predictive Value (Optimal)	Negative Predictive Value (Optimal)	Area Under ROC Curve (95% CI)	*p*-Value
	T/R Ratio							
	Lead							
n = 95	V2	≥0.75 (≥0.48)	0.68 (0.82)	0.80 (0.70)	0.97 (0.96)	0.23 (0.33)	0.80 (0.65–0.94)	<0.01
n = 94	V3	≥0.75 (≥0.43)	0.67 (0.91)	0.60 (0.60)	0.93 (0.95)	0.18 (0.43)	0.73 (0.55–0.92)	0.02
n = 95	V4	≥0.75 (≥0.37)	0.43 (0.73)	0.78 (0.67)	0.95 (0.96)	0.13 (0.21)	0.73 (0.54–0.92)	0.02
n = 92	PR	≥200 ms (≥144 ms)	0.08 (0.86)	1.00 (0.56)	1.00 (0.95)	0.11 (0.29)	0.69 (0.48–0.90)	0.07
n = 95	QRS	≥120 ms (≥82 ms)	0.02 (0.81)	1.00 (0.60)	1.00 (0.95)	0.11 (0.27)	0.71 (0.52–0.90)	0.03
	T/R Ratio							
	Lead							
n = 466	V2	≥0.75 (≥0.57)	0.67 (0.76)	0.58 (0.52)	0.93 (0.93)	0.17 (0.20)	0.68 (0.61–0.76)	<0.01
n = 472	V3	≥0.75 (≥0.58)	0.64 (0.75)	0.60 (0.56)	0.93 (0.94)	0.16 (0.22)	0.68 (0.59–0.76)	<0.01
n = 465	V4	≥0.75 (≥0.51)	0.39 (0.55)	0.77 (0.70)	0.94 (0.94)	0.12 (0.15)	0.62 (0.53–0.71)	<0.01
n = 457	PR	≥200 ms (≥144)	0.08 (0.85)	0.94 (0.35)	0.91 (0.92)	0.10 (0.21)	0.55 (0.46–0.65)	0.23
n = 469	QRS	≥120 ms (≥92)	0.11 (0.49)	1.00 (0.77)	1.00 (0.95)	0.10 (0.15)	0.67 (0.59–0.74)	<0.01

Legend: values of N are slightly different because outliers were excluded using Z-scores.

## Data Availability

The data that support the findings of this study are available on request from the corresponding author (L.S.).
